# (Mal)Adaptive Psychological Functioning of Students Utilizing University Counseling Services

**DOI:** 10.3389/fpsyg.2017.00403

**Published:** 2017-03-15

**Authors:** Valeria Biasi, Rita Cerutti, Luca Mallia, Francesca Menozzi, Nazarena Patrizi, Cristiano Violani

**Affiliations:** ^1^Department of Education, “Roma Tre” UniversityRome, Italy; ^2^Department of Dynamic and Clinical Psychology, Sapienza University of RomeRome, Italy; ^3^Department of Movement, Human and Health Sciences, State University of Rome “Foro Italico”Rome, Italy; ^4^Department of Psychology, Sapienza University of RomeRome, Italy

**Keywords:** university students, counseling, discriminant analysis, Adult Self Report, help-seeking

## Abstract

**Background:** University students confront psychological difficulties that can negatively influence their academic performance. The present study aimed to assess several areas of adaptive and maladaptive psychological functioning among university students who request counseling services.

**Method:** One hundred eighty-four young female students seeking professional psychological help (Counseling seekers) and 185 young female students who have never asked for psychological help (Non-counseling seekers) were asked to complete the Adult Self-Report (ASR) to evaluate both their internalizing and externalizing problems through DSM-oriented scales as well as their adaptive functioning.

**Results:** ANOVA results indicated worse psychological functioning for the students who sought counseling. They reported lower score in ASR Adaptive Functioning Scales (i.e., friends, jobs, family, education), and higher scores in DSM-oriented scales (i.e., Depressive, Anxiety, Somatic, Avoidant Personality, Attention Deficit/Hyperactivity symptoms) than the students who never asked psychological help. Furthermore, discriminant analysis successfully discriminated between the two groups of students on the basis of the ASR’s adaptive and DSM-oriented scales.

**Conclusion:** The study findings could be useful to guide university counseling services in their screening activities as well as useful for clinical practice.

## Introduction

In recent years, research on student access to university primary care services has revealed an unprecedented number of students in psychological distress. A significant increase in mental health issues has been found in this population (Gallagher, 2008), with some studies revealing that approximately half of the university students report moderate levels of stress-related mental health concerns, including anxiety and depression (Bayran and Bilgel, 2008; Garlow et al., 2008; Keyes et al., 2012). Consequently, depression and other mental health disorders among university students represent a significant public health problem, as they can negatively affect the ability of students to adapt, their development and their academic performance (Mowbray et al., 2006; Mackenzie et al., 2011). In this context, university counseling services can play a key role in improving students’ wellbeing and adaptation. An overall assessment of students’ functioning, including educational and social domains and adaptation in addition to mental health problems, can be helpful for planning prevention programs for a large number of students.

A number of factors may contribute to the onset of psychopathological symptoms, as well as psychological adjustment problems during the university years. The transition to university is generally marked by complex changes in emotional, social, and academic adjustment for students, and these changes add stress to the multiple challenges that arise in late adolescence and emerging adulthood development (Hermann et al., 2014). Such concerns involve developing a personal identity, seeking connectedness among peers and developing autonomy from the family of origin (Hinkelman and Luzzo, 2007). Arnett (2000) proposed that emerging adulthood, the period from approximately 18 to 25 years of age, is distinct from both adolescence and adulthood, and is generally characterized by an exploration of life’s possibilities independently of social roles and normative expectations. In this sense, university students encounter additional difficulties, that depend partly on the developmental stage and partly on the adaptation to new environments and lifestyles. Furthermore, mounting psychological conflicts that emerge in various domains can increase stress levels and negatively influence academic functioning. This accumulation of psychological problems generally results in increased levels of mental rigidity, intolerance of incongruity and uncertainty, distress, anxiety, anger, and sadness, which are mental health risks both in childhood and in adolescence and adulthood (Biasi and Bonaiuto, 2012; Biasi et al., 2015; Menozzi et al., 2016).

An ecological-developmental perspective recognizes environmental influences from micro- to macro-systems on students’ developmental processes (Taylor, 2008). Moreover, the ecological model emphasizes that mental health outcomes are the effects of multiple factors operating at multiple levels (Byrd and McKinney, 2012). In this sense, university students encounter additional difficulties that depend partly on the developmental stage and partly on the adaptation to new environments and lifestyles. In a meta-analysis of the scientific literature, Richardson et al. (2012) highlighted five main domains that influence student academic performance: personality traits, motivation factors, self-regulatory learning strategies, the student’s approach to learning, and psychosocial contextual influences. In particular, the authors noted that effort regulation and academic self-efficacy are important correlates of academic performance, while procrastination is negatively correlated with academic performance. Furthermore, the overloading of psychological problems that may emerge in different domains could aggravate stress levels and have a negative impact on academic functioning. An overload of developmental tasks, challenges and psychological conflicts, which generally increase the levels of mental rigidity and intolerance of incongruity and uncertainty, can result in higher levels of distress and anxiety, both increasing the risk for wellbeing and academic success (Biasi et al., 2015, 2016).

Personal and emotional adjustment problems may also negatively influence academic adjustment and student performance. For example, McKenzie and Schweitzer (2001) found a negative association between anxiety and academic performance in first-year Australasian University students as well as a positive association between depression and dropping out of the academic system. Furthermore, the profound reorganization that university students must manage in their academic skills and in their personal and social development can amplify pre-existing psychological distress. Many studies have indicated that anxiety, depression and substance abuse are major problems among college students (Eisenberg et al., 2007b; Hinkelman and Luzzo, 2007; Michael, 2014). In a study of mental health among college students, Kirsch et al. (2015) found that in a sample of students who were referred to a psychiatrist for evaluation and treatment, approximately half of the students had a primary diagnosis of depressive disorder and 55% reported a history of suicidal thoughts. In a systematic review of research on depression prevalence in university students, Ibrahim et al. (2013) noted that the average depression prevalence is 30.6%, which is higher than that found in the general population. Depression is a risk factor for suicide and other adverse experiences. Mackenzie et al. (2011) found a prevalence of suicidal ideation higher than 10% among students accessing university health services and a prevalence higher than 30% among depressed students. A longitudinal study aimed at monitoring the psychological wellbeing of more than 4,000 students throughout their first university year emphasized that anxiety was more prevalent than depression, especially at the beginning of the year (Cooke et al., 2006). This study also showed that the anxiety level reported by students tends to decrease during the first year even if it does not return to the level preceding the beginning of their university career. Furthermore, several studies have reported that the number of students with significant psychological problems requiring counseling services has been increasing in recent decades (Erdur-Baker et al., 2006; Dogan, 2012; Kirsch et al., 2015).

Other studies have focused on the role of risk factors that could influence and amplify the emotional distress experienced by students. Some studies, for example, have addressed the role of psychological problems encountered during childhood (Reef et al., 2010), the presence of past maltreatment or psychopathology (Southerland et al., 2009; Porche et al., 2011), and family problems such as parent job loss or divorce (Cavanagh et al., 2006) as factors increasing the likelihood of distress for student. These findings highlight the vulnerability of university students and the need to improve prevention programs and mental health services to reduce negative effects on their academic performance and their development. Gender is also a factor that may influence students’ psychological wellbeing and their adaptation to university life. Despite the lack of clear empirical evidence, some studies have noted that the university experience may be more challenging and distressing for female students than for males (Lucas and Berkel, 2005), and other studies have found higher levels of depressive symptoms for female students than for males (Hyun et al., 2006; Miller and Prosek, 2013). Furthermore, female students tend to seek professional psychological help more frequently than males do (e.g., Cooke et al., 2006). The prevalence of females among students seeking psychological help from university counseling services has also been confirmed by Italian national studies showing that females seeking help from university counseling services report higher levels of psychopathological symptoms than males (e.g., Monti et al., 2013).

In summary, a considerable number of university students, especially females, experience psychological problems that can interfere with their academic functioning. Only a limited number of students actively seek professional psychological help or counseling services (Komiya et al., 2000). This pattern may be due to the developmental tasks of emerging adulthood, particularly the need for independence, which can interfere with the recognition and disclosure of personal psychological difficulties and prevent help-seeking behaviors. A large survey of university students (Eisenberg et al., 2007a) found that many students with positive screens for depression and anxiety (from 37 to 84%, depending on the disorder) did not receive any psychological help, and that the predictors for not receiving help included a lack of perceived need, lack of awareness of the existence of services or insurance coverage, skepticism about treatment effectiveness, and a background of low socioeconomic status. More recently, Hunt and Eisenberg (2010) reviewed studies addressing mental health problems and help-seeking behaviors in college studies and confirmed that untreated mental disorders are frequent in college students. This result is consistent with findings from population-based studies on requests for psychological help by young adults. Additionally, Vanheusden et al. (2008) assessed internalizing and externalizing problems in approximately 2,000 young adults aged 19–32 using the Adult Self Report (ASR) (Achenbach and Rescorla, 2003) and found that only 34.5% of young adults with clinical levels of psychopathology had used counseling or mental health services. Young adults with clinical levels of psychopathology who did not seek help did not recognize that they had such problems and had a negative perspective on help-seeking (Vanheusden et al., 2008). Moreover, Kearns et al. (2015) administered to 493 Irish university students an on-line survey including measures of stigma of suicide, group identification and experience with help-seeking. The results showed that students who identified more strongly with their university demonstrated a higher stigma for seeking help from their university mental health service. These findings underline the relevance of screening for psychological distress among college students and the importance of providing clinical services beginning with university counseling services to overcome barriers to care and to develop secondary and tertiary prevention programs (Eisenberg et al., 2009).

Overall, assessing the psychological functioning of students approaching university counseling services is necessary to guide these services toward a more efficient organization of their screening activities, clinical practices, and prevention programs. More specifically, assessment of the psychological functioning of students may be important from a multifactorial perspective given the complex interplay between the adaptive and maladaptive functioning among university students. Finally, given the overrepresentation of females among students seeking professional psychological help, focusing on this student population may be of specific interest. Consistent with these general considerations, the present study aimed to assess the adaptive and maladaptive functioning of female university students approaching university counseling services through validated, multifaceted instruments. Specifically, the study considered several adaptive and problematic functioning areas to compare this group of help-seekers with a comparable group of university students who had never sought psychological help. Finally, the study aimed to identify the optimal linear combination of these mal(adaptive) functioning areas to better identify help-seeking students within the university community. These data may be useful for helping and guiding clinical and preventive interventions within the university student community. In contributing to the literature, the present study investigates both individual adaptive and maladaptive functioning using a multivariate approach to analyze their interaction in students seeking formal help.

## Materials and Methods

### Participants and Procedures

The present study compared data from students who sought professional psychological help at the university psychological counseling services (“Counseling seekers” group) with data from students who had never sought professional psychological help or counseling (“Non-counseling seekers” group) in two large public universities in the central area of Rome. All female students who sought counseling during the 2010–2013 academic years completed a questionnaire, the ASR (Achenbach and Rescorla, 2003) that assessed various areas of psychological functioning. Overall, the questionnaires completed by 184 young female students under the age of 30 years (*M* = 23.58; *SD* = 2.79) were considered for the “Counseling seekers” group. The control group consisted of female students under the age of 30 who were contacted on campus through a convenience sampling and were asked to collaborate voluntarily in a study on psychological wellbeing. Overall, 208 female students who had never sought psychological help were contacted after class tests in the Education and Psychology courses, and were asked to participate in the study. One-hundred and eighty-five female students aged 18–29 (*M* = 21.22; *SD* = 2.36) agreed to participate in the study (attrition rate = 11.06%) and gave their consent before completing the questionnaire. This study was conducted in accordance with the Helsinki Declaration as revised in 1989, and it was approved by the Ethics Committees of the principal investigators.

### Measures

Internalizing and externalizing problems as well as adaptive functioning were measured through the Italian version (Ivanova et al., 2014) of the ASR (Achenbach and Rescorla, 2003), a questionnaire that was developed for people aged 18–59 years and that is included in the Achenbach System of Empirically Based Assessment (ASEBA). This system facilitates the assessment of many areas of individual functioning, such as adaptive functioning and problems throughout the lifespan. The ASR comprises two different sections. The items in the first section refer to Adaptive Functioning Scales and provide a global estimate of the respondent’s adaptive functioning. Overall, these items cover adaptive functioning areas such as one’s friends, spouse or partner, family, job, and education. The second section of the ASR consists of 123 items that evaluate behavioral, emotional, and social problems and form eight empirically based syndrome scales (Anxious/Depressed, Withdrawn, Somatic Complaints, Thought Problems, Attention Problems, Aggressive Behavior, Rule-breaking Behavior and Intrusive). The scores for some of these syndrome scales can be added to obtain a score for two broad groups of problems defined as Internalizing (i.e., Anxious/Depressed, Withdrawn, and Somatic Complaints) and Externalizing (i.e., Aggressive Behavior, Rule-breaking Behavior, and Intrusive Behavior). In particular, the present study used the DSM-oriented ASR scales comprising ASR items that experts from many cultures have identified as being highly consistent with the DSM-IV categories (Achenbach and Rescorla, 2003; Achenbach et al., 2005) and that have previously been used in the Italian context (Lombardo et al., 2013): Depressive Problems, Anxiety Problems, Somatic Problems, Avoidant Personality Problems, Attention Deficit/Hyperactivity Problems, and Antisocial Personality Problems. Past studies have confirmed the validity and reliability of the ASR (Achenbach and Rescorla, 2003; Achenbach et al., 2005). In particular, Achenbach and Rescorla (2003) showed how the Adaptive Functioning Scales have an acceptable level of test-retest reliability and internal consistency (*r* = 0.82 and α = 0.69 for friends, *r* = 0.85 and α = 0.78 for spouse/partner, *r* = 0.74 for family, *r* = 0.71 and α = 0.60 for job, and *r* = 0.80 and α = 0.51 for education, respectively). With respect to the DSM-oriented scales, Achenbach et al. (2005) also showed good internal consistency for all of the scales, namely, Depressive Problems (α = 0.79), Anxiety Problems (α = 0.71), Somatic Problems (α = 0.74), Avoidant Personality Problems (α = 0.69), Attention Deficit/Hyperactivity Problems (α = 0.80), and Antisocial Personality Problems (α = 0.76). The reliability coefficients (Cronbach’s alphas) for the Adaptive Functioning Scales and the DSM-oriented scales, calculated for the present sample, are reported in **Table 1**. Overall, the reliability coefficients are consistent with previous research.

**Table 1:** Mean scores on the Adult Self-Report (ASR) Scales between Groups (“Counseling seekers” vs. “Non-counseling seekers”).

	Counseling seekers		Non-counseling seekers		*F*	*P*-level	Eta square	Cronbach’s alpha
	Mean	*SD*	Mean	*SD*				
**Functioning Adaptive Scales**								
Friends	8.28	2.56	9.29	1.67	20.35	<0.001	0.053	0.51
Partner	3.67	2.96	4.85	3.39	1.36	0.249	0.030	0.72
Family	1.25	0.50	1.42	0.47	11.17	0.001	0.030	0.65
Job	0.79	2.21	1.74	1.89	7.06	0.009	0.052	0.52
Education	1.22	2.27	3.80	1.65	129.51	<0.001	0.289	0.71
**DSM-Oriented Scales**								
Depressive Problems	12.11	5.26	5.86	4.2	159.29	<0.001	0.303	0.85
Anxiety Problems	9.40	2.86	6.90	2.77	72.77	<0.001	0.165	0.70
Somatic Problems	4.03	3.31	2.99	2.99	9.98	0.002	0.026	0.75
Avoidant Personality Problems	6.02	3.17	3.41	2.50	76.85	<0.001	0.173	0.78
Attention Deficit/Hyperactivity Problems	9.82	4.49	6.58	3.81	55.82	<0.001	0.132	0.76
Antisocial Problems	4.68	3.09	4.05	4.05	2.88	0.091	0.008	0.68

### Data Analysis

One-way analyses of variance (ANOVAs) were performed to assess the differences in the Adaptive Functioning Scales and DSM-oriented scales across the two groups of students considered in the study (“Counseling seekers” vs. “Non-counseling seekers”). Furthermore, multiple discriminant analysis (stepwise method) was conducted to evaluate the optimal combination of the aforementioned ASR adaptive and DSM-oriented scales that can significantly discriminate between students seeking psychological counseling and the control group. According to the suggestions of the ASR authors (Achenbach and Rescorla, 2003), the ANOVAs and discriminant analysis were performed on raw scale scores to account for the full range of variation in these scales. All analyses were performed using the IBM SPSS software version 21.0. Notably, students who did not have a partner or a job did not respond to the corresponding ASR questions. Consequently, given the small number of valid responses on these two scales (*n* = 45 and *n* = 129 for the partner and job scales, respectively), they were not used for the discriminant analysis.

## Results

### Adaptive and DSM-Oriented Scale Scores in Counseling Seekers and Non-counseling Seekers

**Table 1** reports the means across the groups for each scale considered. The ANOVAs conducted on the ASR Adaptive Functioning Scales showed statistically significant differences in the scales related to friends [*F*_(1,367)_ = 20.35; *p* < 0.001], job [*F*_(1,128)_ = 7.06; *p* = 0.009], family [*F*_(1,367)_ = 11.17; *p* = 0.001], and education [*F*_(1,367)_ = 129.51; *p* < 0.001]. Comparing the means of the two groups, one can clearly observe that the differences are consistent with the expectations of the study, as the young female students seeking psychological help from a counseling service obtained the lowest scores in all functioning areas, including friends, family, and education, with the exception of the sentimental domain [partner scale, *F*_(1,44)_ = 1.36; *p* = 0.256]. Furthermore, analysis of the effect size reported in **Table 1** clearly indicates that the greatest differences between the two groups concern the educational domain (η^2^ = 0.29), with lower levels reported by the female students seeking psychological help compared with those not seeking help.

With respect to the ANOVAs of the ASR DSM-oriented scales, the results show statistically significant differences for the scales assessing Depressive Problems [*F*_(1,367)_ = 159.29; *p* < 0.001], Anxiety Problems [*F*_(1,367)_ = 72.77; *p* < 0.001], Somatic Problems [*F*_(1,367)_ = 9.98; *p* = 0.002], Avoidant Personality Problems [*F*_(1,367)_ = 76.85; *p* < 0.001], and Attention Deficit/Hyperactivity Problems [*F*_(1,367)_ = 55.82; *p* < 0.001]. No statistically significant effect emerged for the Antisocial Problems scale [*F*_(1,367)_ = 2.88; *p* = 0.091]. Overall, the means reported in **Table 1** clearly show that counseling-seeking students obtain higher scores in most of the problematic areas assessed by the ASR compared to the non-counseling seekers. Furthermore, an analysis of effect sizes reveals the most marked between groups difference in the area of Depressive problems (η^2^ = 0.30), while the differences for Avoidant Personality, Anxiety, and Attention Deficit and Hyperactivity Problems are less relevant (η^2^ = 0.17, 0.16, and 0.13, respectively).

### Discriminant Analysis

The results of the discriminant analysis are summarized in **Tables 2, 3**. Only one discriminant function was extracted to distinguish the two groups considered in the study [Wilkin’s Lambda_(6)_ = 0.53; *p* < 0.001]. This function represents the linear combinations of the predictors included in the analysis and creates a new latent variable maximizing the differences between groups. The canonical correlation for the function extracted is acceptable (0.68); thus, the model explains the significant relationship between the function and the dependent grouping variables. Discriminant analysis using the stepwise method included only six variables in the discriminant function. **Table 2** reports the standardized coefficients for each variable included in the function, indicating the relative importance of each variable within the discriminant function, particularly its importance in predicting group assignment. The standardized coefficients suggest that Depressive Problems (-0.71) and education functioning (0.68) were the two variables that contributed most to discriminating between the two groups of students, followed by Anxiety Problems (-0.49) and the variable measuring functioning with friends (0.30). Finally, **Table 3** reports the classification matrix of the discriminant analysis results for the cases of the two groups which were correctly classified on the basis of the discriminant function. Overall, as reported in **Table 3**, the discriminating power is good since, based on the discriminant function extracted, more than 80% of the students were correctly assigned to their original group. The highest rate of correct classifications (87.4%) was observed for the counseling-seeking students, while more than 3/4 of the non-seekers (78.7%) were correctly re-assigned to their group.

**Table 2:** Results of the discriminant analysis.

ASR scale loadings	Function 1
Depressive Problems	-0.708
Education	0.677
Anxiety Problems	-0.495
Friends	0.299
Somatic Problems	-0.176
Antisocial Personality Problems	-0.081
**Groups Centroids**	
Counseling Seekers	-0.840
Non-counseling Seekers	1.040
Eigenvalue	0.88
Canonical Correlation	0.68
Chi Square	198.825
Wilk’s Lambda	0.532
Sig.	<0.001

**Table 3:** Classification matrix of the discriminant analysis.

		Groups	Predicted group membership		Total
			Counseling seekers	Non-counseling seekers	
**Original group membership**	%	Counseling seekers	87.4%	12.6%	100%
		Non-counseling seekers	21.3%	78.7%	100%

## Discussion

The present study aimed to assess several areas of adaptive and maladaptive psychological functioning among female university students who request counseling services and among students who have not sought psychological help to define the main characteristics differentiating these groups of students.

The univariate analysis confirmed that female students who request counseling services report poorer psychological functioning considering both adaptive and problematic areas. More specifically, counseling seekers showed greater differences from non-counseling seekers in the areas of educational functioning, as they reported a weak capacity to cope and manage the demands related to their university career. These students also reported lower functioning in the general social domain that affects both family and friend relationships. With respect to the problematic areas, the students approaching university counseling services primarily reported difficulties in emotional regulation areas, obtaining higher scores in the Depressive and Anxiety Problems scales. These results are fully consistent with the findings of studies in other countries (e.g., Eisenberg et al., 2007a; Hinkelman and Luzzo, 2007) showing that anxiety and depression are the main psychological problems among college students. These studies have also emphasized that many students who struggle with depression and anxiety symptoms do not perceive a need for help and do not seek clinical support (Eisenberg et al., 2007b), thus calling for more attention for information and screening by the university counseling services. Notably, high levels of Attention Deficit and Hyperactivity Problems could negatively affect adaptation in the educational domain. Finally, the relevance of social functioning problems among counseling seekers is confirmed by their high scores on the Avoidant Personality scale, indicating difficulty in interpersonal relations that may lead to social isolation and loss of social support.

The multivariate analytic approach more clearly identifies critical areas characterizing female students seeking counseling. In particular, discriminant analysis shows that these students can be identified through a multivariate combination of their scores on the scale related to emotional regulation (i.e., Depressive problem scale), functioning in the university domain (i.e., Educational scale), and interpersonal relationships (i.e., Friends). Overall, these findings are consistent with previous research emphasizing the relevance of emotional regulation problems (i.e., anxiety and depression) in university student populations (e.g., Eisenberg et al., 2007a; Hinkelman and Luzzo, 2007), particularly among female students (Biasi and Bonaiuto, 2014).

With respect to the general aims of the present study, the results discussed above confirm the findings in other countries and may be useful for guiding university counseling services in organizing more efficiently their screening activities and clinical practice. One indication relates to the usefulness of assessing students by focusing on both adaptive functioning and psychological difficulties. Indeed, the discriminant analysis in this study was able to identify students seeking help by combining information on both adaptive and maladaptive psychological functioning. According to these findings, the screening activities used by counseling services should employ assessment instruments including scales that address these two domains rather than instruments focusing only on psychological maladaptive functioning. A second indication is the importance of functioning in the specific domain of education for students approaching university counseling. In fact, along with their psychological difficulties, these students clearly reported a weak capacity to meet the demands of their academic environment. Based on this evidence, it appears crucial for counseling services to improve their capacity to adequately address the difficulties of students with general psychological problems. A third indication is that students approaching counseling services show general difficulties in the relationship domain. Hence, university counseling services should design and implement programs to promote interpersonal skills and to facilitate friendship networks within the university community.

### Limitations

The present study is not without limitations. The main limitation that we wish to address is the cross-sectional design of the study. Future longitudinal studies should replicate the predictive value of our results by following students in their careers with respect to their psychological functioning and help-seeking behaviors. Another limitation is the exclusive reliance on self-report measures of psychopathological symptoms. Additionally, the self-report measures covering adaptive functioning about friends and job revealed a low reliability, calling attention about the possibility of biases in the results related to these areas, that need to be considered with caution. Several reasons can explain this weakness such as the low number of respondents (e.g., as in the case of “Job” scale, that was excluded from the discriminant analysis) and/or bias in the scale’s translation and adaptation procedures. In this sense further studies are needed to replicate the present results, trying to overcome these weaknesses.

Furthermore, it is important to call the attention on the fact that the use of a convenience sample as control group could insert biases, enlarging the differences between the two groups that the present study highlighted. It is plausible that the students who were contacted in the university buildings and accepted to participate have less problems and higher functioning than students who never attend classes. Future studies using randomly selected students as control group are needed in order to control and overcome these possible biases. Additionally, it is to note that the study did not controlled the possible effects of the students’ course years on the key variables of the study. In fact, it is plausible that the adaptation and psychological functioning of the students could vary across the university attendance years. Future studies controlling for this possible confounding variable are need in order to exclude and control possible bias in results.

Finally, this study was conducted in two large highly qualified public universities located in the same city, which may limit the generalizability of the findings to other settings, such as small or private institutions.

## Conclusion

The relevance of the present study is derived from the application of the ASR questionnaire developed by Achenbach and Rescorla (2003) in the assessment of multiple areas of individual functioning, including both adaptive and maladaptive functioning of emotional and relational processes throughout the lifespan. The current study provides further evidence that students approaching university counseling services report not only psychological problems but also specific adaptive difficulties in functioning in the academic domain. The findings confirm the specificity of university counseling services that should be oriented toward addressing not only psychological difficulties but also specific areas of the academic and interpersonal functioning (Thomas and Henning, 2012). A notable strength of this study is the assessment of individual mal(adaptive) functioning using a multivariate approach, which allowed to establish a specific linear combination of the ASR scales to identify students approaching counseling services. Future studies are needed to verify the capacity of this analytical approach to predict students’ help-seeking behaviors from a longitudinal perspective.

## Author Contributions

All the authors substantially have equally contributed to the development and preparation of the manuscript. Furthermore, all authors have approved the final version of the manuscript. Finally, the authors have agreed to be accountable for all aspects of the manuscript in ensuring that questions related to the accuracy or integrity of any part of it are appropriately investigated and resolved.

## Conflict of Interest Statement

The authors declare that the research was conducted in the absence of any commercial or financial relationships that could be construed as a potential conflict of interest.
